# Unsupervised Immune Profiling Identifies Distinct Post-Transplant T-Cell Clusters Associated with Kidney Allograft Function

**DOI:** 10.3390/medsci14020238

**Published:** 2026-05-04

**Authors:** Lampros Vagiotas, Asimina Fylaktou, Ariadni Fouza, Efstratios Kasimatis, Georgios Tsoulfas, Maria Daoudaki

**Affiliations:** 1 Department of Transplant Surgery, Center for Research and Innovation in Solid Organ Transplantation, School of Medicine, Faculty of Health Sciences, Aristotle University of Thessaloniki, 54124 Thessaloniki, Greece; lampisv@yahoo.gr (L.V.); ariadnefou@gmail.com (A.F.); frasci@outlook.com.gr (E.K.); tsoulfasg@auth.gr (G.T.); 2 Department of Immunology, National Peripheral Histocompatibility Center, Hippokration General Hospital of Thessaloniki, 54642 Thessaloniki, Greece; fylaktoumina@gmail.com; 3 Laboratory of Biological Chemistry, School of Medicine, Faculty of Health Sciences, Aristotle University of Thessaloniki, 54124 Thessaloniki, Greece

**Keywords:** CD16/56^+^ T cells, T cells, senescence, graft function, unsupervised clustering

## Abstract

Background: Post-transplant immune heterogeneity may influence kidney allograft outcomes, yet the clinical relevance of circulating T-cell phenotypes remains incompletely defined. We aimed to identify 12-month post-transplant data-driven T-cell clusters and examine their relation to graft-function patterns during the first post-transplant year. Methods: Peripheral blood T-cell subpopulations were analyzed in 112 kidney transplant recipients at 12 months post-transplantation using flow cytometry. Standardized subpopulation frequencies underwent unsupervised hierarchical clustering, with principal component analysis used for visualization. Longitudinal graft function trajectories (eGFR and serum creatinine at 1, 3, 6, and 12 months) were analyzed using generalized estimating equation models, including time-by-cluster interactions. Results: Three recipient clusters were identified: a CD8-skewed cytotoxic/senescent cluster, an innate-like cytotoxic cluster, and a CD4-dominant cluster. Cluster robustness was supported by complementary k-means analysis. Older recipient age and baseline cytomegalovirus seropositivity were associated with the CD8-skewed cluster. Recipients assigned to different T12 clusters showed differences in serum creatinine levels and graft function trajectories, although some associations were attenuated after additional adjustment for transplant-related factors. Conclusions: In this cohort, unsupervised clustering identified distinct post-transplant T-cell profiles associated with early graft function patterns. These findings are hypothesis-generating and require longitudinal and external validation.

## 1. Introduction

Kidney transplantation represents the optimal treatment for end-stage renal disease, providing substantial improvements in survival and quality of life compared with dialysis [1,2]. Nevertheless, long-term graft survival remains suboptimal, with progressive deterioration of renal function frequently occurring even in the absence of overt clinical rejection [3,4]. Conventional clinical and transplant-related risk factors only partially explain variability in graft outcomes, reflecting the need for improved biological markers of graft health [5,6,7,8].

T-cells play a central role in alloimmune responses after transplantation, contributing to both acute rejection and chronic immune-mediated graft injury [9,10]. Increasing evidence indicates that alterations in circulating T-cell subsets are associated with graft outcomes [11,12,13,14,15]. However, immune signatures derived from individual cell populations may not adequately capture the complexity of post-transplant immune adaptation [16,17]. Most immunomonitoring approaches rely on predefined cell populations or isolated biomarkers assessed cross-sectionally [18].

In contrast, data-driven unsupervised analytical approaches, including clustering and principal component analysis, enable characterization of the multidimensional immune architecture [19] and identification of biologically coherent immune profiles [20,21]. Integrating immune profiling with longitudinal assessment of graft function may uncover clinically relevant associations not apparent in traditional cross-sectional analyses [5,22].

In this study, we applied unsupervised clustering and principal component analysis of circulating T-cell subsets measured 12 months after kidney transplantation to identify recipient clusters with distinct T-cell composition patterns and to examine their association with kidney allograft function trajectories during the first post-transplant year. Because immune profiling was performed at 12 months post-transplantation, analyses were designed to assess retrospective associations with graft function patterns during the first year rather than prospective prediction.

## 2. Materials and Methods

### 2.1. Study Population

This prospective cohort study included adult kidney transplant recipients who completed the 12-month post-transplant assessment. Participants were evaluated at the time of transplantation (T0) and at one (T1), three (T3), six (T6), and twelve months (T12) after transplantation.

Inclusion criteria comprised adults aged 18–70 years with regular nephrology follow-up for at least 2 years prior to transplantation.

Exclusion criteria included history of malignancy, autoimmune disease, or hematological disease; treatment with monoclonal antibodies against B- or T-lymphocytes within the preceding 5 years; active cytomegalovirus or bacterial infection within 3 months before enrolment; unexplained deterioration of renal function; follow-up <2 years; recipients from deceased donors with cardiac arrest; and non-adherence to treatment instructions.

A total of 125 recipients were initially enrolled. Following withdrawals and exclusions due to missing records, relapse of primary disease, or infection during follow-up, the final study cohort consisted of 112 recipients with available T12 immunophenotyping data.

### 2.2. Clinical Data and Study Assessments

Demographic and clinical data were extracted from medical records (Table 1). Blood samples for laboratory and immunological evaluation were collected at all predefined study time points. Renal function was assessed using estimated glomerular filtration rate (eGFR) calculated with the CKD-EPI 2021 equation.

No preformed donor-specific anti-HLA antibodies (DSA) were detected in any recipient prior to transplantation. All transplants were performed under ABO-compatible conditions with a negative crossmatch.

### 2.3. Ethics Approval and Consent to Participate

The study was conducted in accordance with the Declaration of Helsinki and approved by the Institutional Review Board of the Medical School of the Aristotle University of Thessaloniki (ref. no. 2348/24 November 2020). Written informed consent was obtained from all participants.

### 2.4. Immunosuppression Regimen

Immunosuppressive therapy followed the institutional protocol, including risk-adapted induction and standard triple maintenance therapy. Induction therapy consisted of an anti-CD25 antibody (basiliximab), administered in 95 recipients at 20 mg pre-transplant and on postoperative day 4 together with perioperative methylprednisolone (500 mg) in standard-risk patients, whereas 17 recipients with increased immunological risk received anti-thymocyte globulin (ATG, 1.5 mg/kg/day for 4 days; cumulative dose ≈ 6 mg/kg). Maintenance immunosuppression consisted of tacrolimus (target trough 6–8 ng/mL during the first year), mycophenolate mofetil (2 g/day initially, reduced to 1 g/day), and corticosteroids administered as intravenous methylprednisolone followed by oral tapering until day 42. Rejection episodes were treated with anti-thymocyte globulin (ATG) for T cell–mediated rejection and corticosteroids, plasma exchange, and ± intravenous immunoglobulin for antibody-mediated rejection.

### 2.5. Flow Cytometry of T-Cell Subpopulations

Peripheral blood T-cell subsets were quantified at T12 using flow cytometry (Navios EX flow cytometer, Beckman Coulter, Brea, CA, USA). The following populations were analyzed: CD3^+^CD4^+^, CD3^+^CD4^+^CD28^+^, CD3^+^CD4^+^CD28^−^, CD3^+^CD8^+^, CD3^+^CD8^+^CD28^+^, CD3^+^CD8^+^CD28^−^, CD3^+^CD16^+^/56^+^ cells, and regulatory T cells (Tregs). Whole blood samples were processed immediately after collection and stained with fluorochrome-conjugated monoclonal antibodies (anti-CD45, anti-CD3, anti-CD4, anti-CD8, anti-CD16, anti-CD56, and anti-CD28), as previously described [23]. The immunophenotyping panel focused on clinically available markers of circulating T-cell subsets (CD4, CD8, CD28, CD16/56, and FOXP3-defined Tregs). Markers of activation, exhaustion, and detailed naïve/memory differentiation (e.g., HLA-DR, CD69, PD-1, TIM-3, CCR7, and CD45RA) were not included. The cluster labels were interpreted as descriptive immunophenotypic patterns, with the terms ‘senescent/cytotoxic’ and ‘innate-like’ used to refer to the dominant immunophenotypic characteristics within each group, rather than as a functional classification.

#### Treg Identification (Intracellular FOXP3 Staining)

Regulatory T cells were defined as CD4^+^CD25^+^FOXP3^+^. Peripheral blood mononuclear cells were isolated by Ficoll density-gradient centrifugation, followed by surface staining, fixation, permeabilization, and intracellular FOXP3 staining [23]. Treg frequencies were reported relative to CD4^+^ T cells.

### 2.6. Immune Profiling and Unsupervised Analysis

Standardized (z-score transformed) T-cell subset frequencies measured at T12 were used for immune profiling. Unsupervised hierarchical clustering was performed using Ward’s minimum variance method (Ward.D2) with Euclidean distance to identify groups of recipients [24] with similar multidimensional T-cell profiles. The number of clusters was evaluated across candidate solutions (k = 2–5) using silhouette coefficients.

Cluster robustness was assessed using k-means clustering as a complementary partitioning approach applied to the same standardized variables, with the number of clusters fixed at k = 3 for direct comparison with the primary hierarchical solution. Agreement between methods was evaluated descriptively. Detailed clustering metrics are provided in the Supplementary Material.

The T12 time point was selected a priori to characterize immune composition after the early peri-transplant inflammatory phase and after stabilization of maintenance immunosuppression, thereby reducing the influence of transient early post-transplant perturbations. The identified groups were interpreted as exploratory cluster-defined patterns within this cohort rather than externally validated biological immune entities. PCA was applied for dimensionality reduction and visualization of cluster separation.

**Figure 1:**
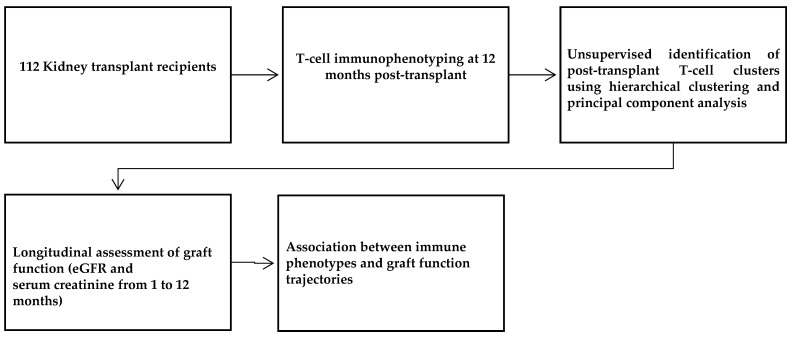
Study workflow illustrating immune stratification of kidney transplant recipients at 12 months post-transplantation.

The overall analytical workflow is summarized in Figure 1.

#### Clinical and Transplant-Related Variables

Clinical and transplant-related variables were examined across immune clusters to assess their potential associations with graft function. Variables included recipient age, sex, duration of dialysis prior to transplantation, donor type (living or deceased), cold ischemia time, occurrence of delayed graft function, acute rejection episodes, induction therapy, and baseline cytomegalovirus (CMV) IgG serostatus at T0.

Variables included in subsequent regression analyses were selected a priori based on biological plausibility, clinical relevance, data completeness, and previous evidence linking these factors to transplant outcomes or post-transplant immune phenotypic patterns.

Categorical variables were analyzed as binary or nominal, while continuous variables were analyzed as continuous measures.

Between immune clusters, comparisons were conducted using statistical tests appropriate to variable type and distribution. Continuous data were presented as median (interquartile range) and categorical data as counts and percentages.

### 2.7. Longitudinal Graft Function Analysis

Graft function was assessed using serial measurements of serum creatinine and eGFR at 1, 3, 6, and 12 months post-transplantation. Associations between clusters and graft function trajectories during the first post-transplant year were evaluated using repeated-measures generalized estimating equation (GEE) models with an exchangeable correlation structure and robust standard errors.

Models included centered time (time centered at 5.5 months), clusters, and a time-by-cluster interaction term. Prespecified adjusted models included recipient age and sex, while sensitivity models additionally adjusted for delayed graft function, acute rejection, donor type, cold ischemia time, and baseline CMV serostatus.

Because clusters were defined at 12 months post-transplantation, longitudinal analyses were interpreted as retrospective associations between cluster assignment and graft function patterns during the first post-transplant year rather than prospective prediction or causal effects. This approach was chosen to characterize clusters after immune adaptation following the early peri-transplant inflammatory period and the highest intensity of immunosuppressive therapy. Consequently, clusters defined at T12 were interpreted as reflecting integrated immune phenotypic patterns during the first post-transplant year rather than predictors of subsequent outcomes.

### 2.8. Statistical Analysis

Continuous variables are presented as medians with interquartile range (IQR), and categorical variables as counts and percentages. Comparisons across clusters were performed using the Kruskal–Wallis test for continuous variables and the chi-squared or Fisher’s exact test for categorical variables, as appropriate. When applicable, pairwise post hoc comparisons were conducted with adjustment for multiple testing. Spearman’s rank correlation coefficient was used to assess associations between continuous variables. A two-sided *p*-value < 0.05 was considered statistically significant.

Longitudinal associations between clusters and graft function were evaluated using generalized estimating equation (GEE) models, including centered time, clusters, and a time-by-cluster interaction term. Recipient age and sex were included as prespecified covariates. Sensitivity models were additionally adjusted for delayed graft function, acute rejection, donor type, cold ischemia time, and baseline CMV IgG serostatus. Because the GEE analyses were prespecified exploratory models evaluating two longitudinal outcomes, no formal multiplicity correction was applied; *p*-values were interpreted cautiously and in the context of effect sizes and consistency across models.

Clinical determinants of cluster assignment were evaluated using multinomial logistic regression analysis, using the CD4-dominant cluster as the reference category. Results are presented as odds ratios with 95% confidence intervals. All analyses were performed using R software (version 4.3).

#### Clinical Variables Associated with Cluster Assignment

To explore whether baseline demographic and transplant-related characteristics were associated with post-transplant T-cell cluster assignment, multinomial logistic regression analysis was performed. Post-transplant T-cell cluster assignment (CD4-dominant, CD8-skewed senescent/cytotoxic, and innate-like cytotoxic clusters) was used as the dependent variable, with the CD4-dominant cluster serving as the reference category.

Covariates were selected a priori based on biological plausibility, clinical relevance, and previous evidence linking these factors to transplant outcomes or post-transplant immune phenotypic patterns. These included recipient age, sex, anti-thymocyte globulin (ATG) induction therapy, donor type (living versus deceased), dialysis vintage prior to transplantation, and baseline CMV serostatus as covariates. Results are reported as odds ratios (OR) with 95% confidence intervals (CI) and corresponding *p*-values. All analyses were performed using R software.

## 3. Results

### 3.1. Study Population

A total of 112 kidney transplant recipients with available 12-month immunophenotyping data were included. Baseline clinical and transplant-related characteristics are summarized in Table 1.

During the first post-transplant year, 10 recipients (8.9%) experienced acute rejection, including four cases of T cell–mediated rejection, four cases of mixed T cell– and antibody-mediated rejection, and two cases of antibody-mediated rejection.

Recipient age differed across clusters (*p* = 0.039), whereas sex, donor type, dialysis vintage, delayed graft function, and rejection episodes were comparable between groups Table 1.

### 3.2. Identification of Post-Transplant T-Cell Clusters at 12 Months

Unsupervised hierarchical clustering of standardized T-cell subset frequencies at 12 months post-transplantation identified three distinct recipient clusters, comprising 30, 18, and 64 individuals, respectively. Silhouette analysis across k = 2–5 supported the three-cluster solution as optimal, with the highest silhouette coefficient observed for k = 3 (Supplementary Table S1 and Figure S1).

Projection of cluster assignments onto principal component analysis (PCA) space demonstrated separation primarily along the first principal component, driven by relative contributions of CD4^+^ and CD8^+^ compartments, with additional contribution from CD3^+^CD16^+^/56^+^ T-cell frequencies. Cluster stability was supported by complementary k-means clustering, with 83.04% agreement between hierarchical and k-means assignments (Supplementary Table S2).

The clusters were defined as the following:

- A CD8-skewed senescent/cytotoxic cluster, characterized by enrichment of CD8^+^ and CD8^+^CD28^−^ T cells, cluster 1
- An innate-like cytotoxic cluster, enriched in CD3^+^CD16^+^/56^+^ T cells, cluster 2
- A CD4-dominant helper cluster, characterized by higher CD4^+^ and CD4^+^CD28^+^ T-cell frequencies, cluster 3.

**Figure 2:**
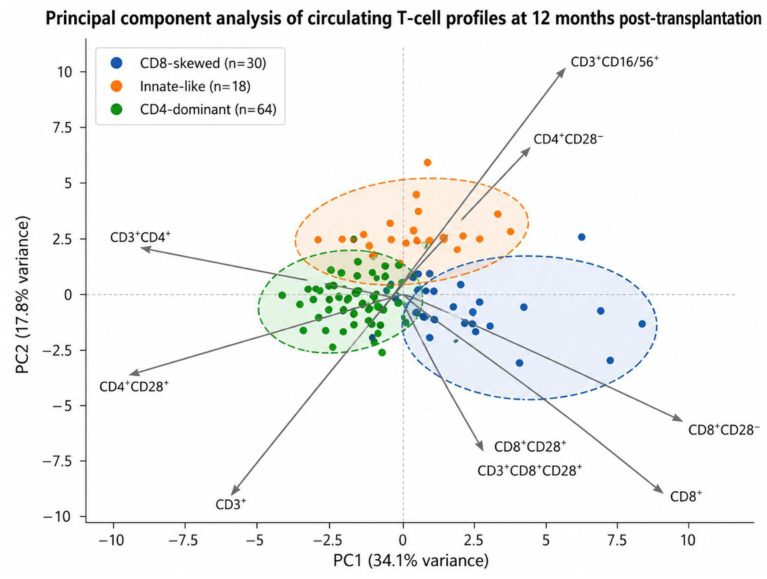
Identification of post-transplant T-cell clusters.

These cluster-defined profiles showed limited overlap in PCA space, supporting biological separation, Figure 2.

Principal component analysis (PCA) of standardized frequencies of circulating T-cell subpopulations in kidney transplant recipients at 12 months post-transplantation. Individuals are colored according to cluster assignment derived from unsupervised hierarchical clustering using Ward’s minimum variance method (Ward.D2 linkage with Euclidean distance, k = 3). Cluster 1 (n = 30) showed a CD8-skewed senescent/cytotoxic profile, Cluster 2 (n = 18), an innate-like cytotoxic profile enriched in CD3^+^CD16^+^/56^+^ T cells, and Cluster 3 (n = 64), a CD4-dominant profile. Arrows indicate loadings of individual T-cell subpopulations on the principal components and their contribution to cluster separation. The first two principal components explained 34.1% (PC1) and 17.8% (PC2) of the total variance, respectively. Ellipses indicate 95% confidence regions for each cluster.

**Figure 3:**
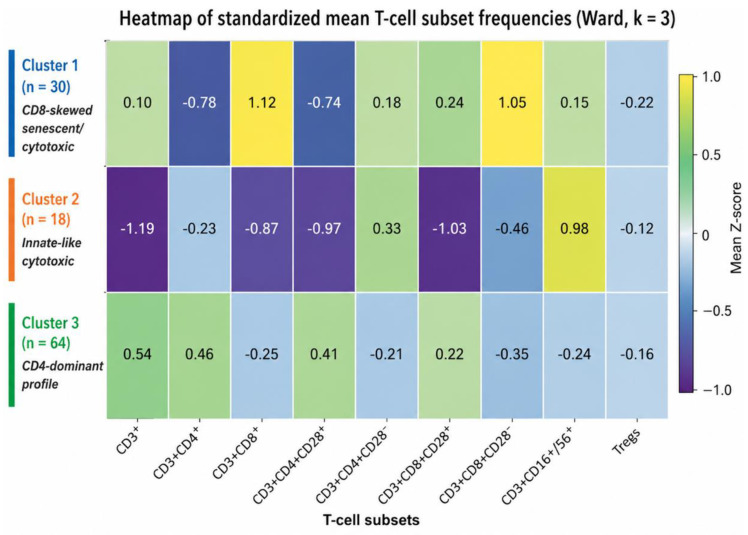
Heat map of post-transplant T-cell clusters at 12 months.

Heat map visualization further illustrated the coordinated T-cell subset patterns across clusters (Figure 3). The CD8-skewed senescent/cytotoxic cluster showed relative enrichment of differentiated CD8^+^ and CD8^+^CD28^−^ T-cell compartments, whereas the CD4-dominant cluster showed enrichment of CD4^+^ and CD4^+^CD28^+^ T-cell subsets. The innate-like cytotoxic cluster was characterized by increased frequencies of CD3^+^CD16^+^/56^+^ T cells. Overall, the heat map illustrates coordinated differences in T-cell subset distribution underlying cluster separation.

Heat map of standardized (z-score) mean frequencies of circulating T-cell subpopulations in kidney transplant recipients at 12 months post-transplantation, displayed according to cluster assignment derived from unsupervised hierarchical clustering. Rows represent the three clusters: CD8-skewed senescent/cytotoxic cluster (Cluster 1, n = 30), innate-like cytotoxic cluster enriched in CD3^+^CD16^+^/56^+^ T cells (Cluster 2, n = 18), and CD4-dominant cluster (Cluster 3, n = 64). Columns represent individual T-cell subpopulations included in clustering. Color intensity indicates relative enrichment (higher z-scores) or depletion (lower z-scores) within each cluster.

### 3.3. Quantitative Immunophenotypic Differences Across Clusters

Quantitative comparison of circulating T-cell subpopulations at 12 months confirmed significant immunophenotypic differences across the three clusters (Table 2). The CD8-skewed senescence/cytotoxic cluster demonstrated higher frequencies of CD3^+^CD8^+^ T cells and CD3^+^CD8^+^CD28^−^ T cells than the innate-like and CD4-dominant clusters (*p* < 0.001 for both). This cluster also exhibited higher CD3^+^CD4^+^CD28^−^ frequencies (*p* = 0.009), consistent with broader CD28 loss across T-cell compartments.

The CD4-dominant helper cluster was characterized by increased CD3^+^CD4^+^ and CD3^+^CD4^+^CD28^+^ T-cell frequencies (*p* < 0.001 for both). In contrast, CD3^+^ frequencies differed across groups (*p* = 0.00023), with lower CD3^+^ values in the innate cluster. The innate-like cytotoxic cluster was distinguished by enrichment of CD3^+^CD16^+^/56^+^ T cells compared with both CD8-skewed and CD4-dominant clusters (*p* = 0.048), while showing lower frequencies of CD3^+^CD8^+^ and CD3^+^CD4^+^CD28^−^ subsets. Regulatory T-cell frequencies did not differ significantly across clusters (*p* = 0.352), indicating that cluster separation was primarily driven by differences in cytotoxic/effector T-cell composition rather than overall regulatory T-cell abundance.

### 3.4. Kidney Function and Transplant-Related Characteristics at 12 Months Across Clusters

Clinical and transplant-related characteristics according to post-transplant T-cell clusters are summarized in Table 3. Recipient age differed significantly across clusters (*p* = 0.039), with older recipients observed in both the CD8-skewed and innate-like cytotoxic clusters compared with the CD4-dominant cluster.

At 12 months post-transplantation, serum creatinine levels showed a numerical gradient across clusters, with higher values observed in the CD8-skewed, intermediate in the innate-like cluster, and lowest in the CD4-dominant cluster, although these differences did not reach statistical significance (*p* = 0.185). Similarly, eGFR values were comparable across clusters (*p* = 0.192). Dialysis vintage and cold ischemia time were comparable across groups (*p* = 0.338 and *p* = 0.645, respectively). The distribution of donor type, sex, delayed graft function, and rejection episodes did not differ significantly across clusters.

Given the absence of statistically significant cross-sectional differences in graft function at 12 months, longitudinal analyses were performed to assess whether cluster assignment was associated with differing graft function patterns during the first post-transplant year.

### 3.5. Longitudinal Graft Function Trajectories Across Clusters (GEE Models)

**Figure 4:**
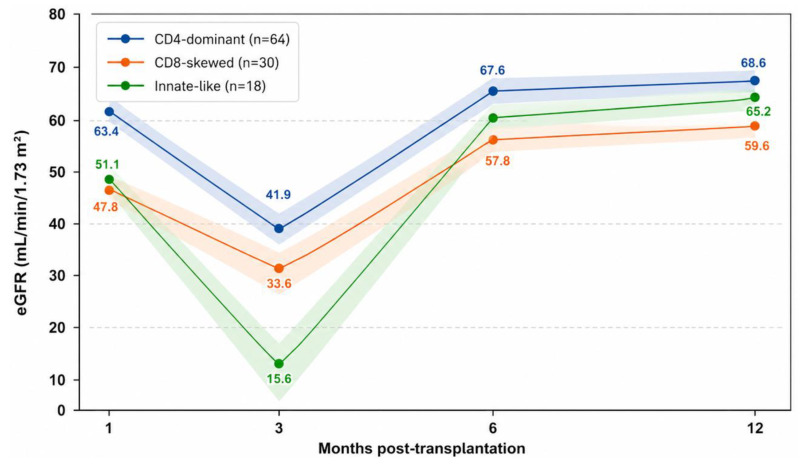
Retrospective eGFR trajectories during the first post-transplant year according to T12 cluster assignment. Lines represent mean values and shaded areas indicate standard error of the mean (SEM).

**Figure 5:**
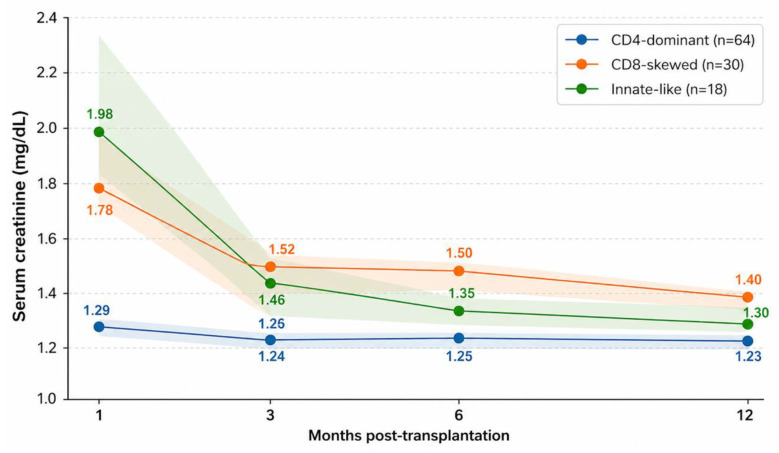
Retrospective serum creatinine trajectories during the first post-transplant year according to T12 cluster assignment. Lines represent mean values and shaded areas indicate standard error of the mean (SEM).

Longitudinal graft function (eGFR and serum creatinine at 1, 3, 6, and 12 months post-transplantation) was analyzed using generalized estimating equation (GEE) models including centered time (centered at 5.5 months), cluster assignment, and a time-by-cluster interaction term, Figure 4 and Figure 5.

#### 3.5.1. eGFR

In the prespecified adjusted model (age and sex), eGFR increased over time (β = +1.25 per month, *p* = 6.0 × 10^−8^). Compared with the CD4-dominant cluster, both the CD8-skewed and innate-like clusters showed numerically lower average eGFR levels, although not statistically significant (β = −8.20, *p* = 0.051 and β = −8.79, *p* = 0.063, respectively). A significant time-by-cluster interaction was observed for the innate-like cluster (β = +1.37, *p* = 0.0015), indicating a different eGFR trajectory relative to the CD4-dominant cluster.

In sensitivity models additionally adjusting for delayed graft function, acute rejection, donor type, and cold ischemia time, between-cluster differences in average eGFR were attenuated, while delayed graft function and acute rejection remained strongly associated with lower eGFR. The time-by-cluster interaction for the innate-like cluster remained significant, Figure 4.

#### 3.5.2. Serum Creatinine

In the prespecified model adjusted for age and sex, the CD8-skewed cluster was associated with higher average serum creatinine compared with the CD4-dominant cluster (β = +0.296 mg/dL, *p* = 0.017), while the innate-like cluster showed a similar direction with a non-significant numerical increase (β = +0.289 mg/dL, *p* = 0.083).

A time-by-cluster interaction was observed for the CD8-skewed cluster (β = −0.020 mg/dL per month, *p* = 0.040), with a numerical trend that did not reach statistical significance for the innate-like cluster (β = −0.052, *p* = 0.052), Figure 5.

In sensitivity models additionally adjusting for transplant-related covariates, the association between the CD8-skewed cluster and higher creatinine remained significant (β = +0.217 mg/dL, *p* = 0.038), whereas the estimate for the innate-like cluster remained numerically elevated but non-significant. Delayed graft function remained independently associated with higher serum creatinine.

Because cluster assignment was based on T12 immunophenotyping, these findings are interpreted as retrospective associations with first-year graft function trajectories rather than predictive effects.

Estimated glomerular filtration rate (eGFR) was measured at 1, 3, 6, and 12 months post-transplantation according to cluster assignment derived from unsupervised hierarchical clustering at T12.

This plot illustrates retrospective associations between T12 cluster assignment and first-year graft function patterns and should not be interpreted as a predictive model.

Serum creatinine was measured at 1, 3, 6, and 12 months post-transplantation according to cluster assignment derived from unsupervised hierarchical clustering at T12.

This plot illustrates retrospective associations between T12 cluster assignment and first-year graft function patterns and should not be interpreted as a predictive model.

### 3.6. Clinical Determinants of Cluster Assignment

To explore whether baseline demographic and transplant-related characteristics were associated with cluster assignment, multinomial logistic regression analysis was performed, Table 4. The CD4-dominant cluster was used as the reference category.

Older recipient age was independently associated with increased odds of belonging to the CD8-skewed senescent/cytotoxic cluster compared with the CD4-dominant cluster (OR 1.05 per year, 95% CI 1.01–1.10, *p* = 0.018). Baseline CMV seropositivity (IgG) was also independently associated with increased odds of a CD8-skewed cluster (OR 7.60, 95% CI 1.67–34.60, *p* = 0.009). No significant associations were observed between the CD8-skewed cluster and recipient sex, donor type, dialysis vintage, or ATG induction therapy. For the innate-like cluster, no independent associations reached statistical significance, although ATG induction showed a non-significant trend (OR 2.91, 95% CI 0.90–9.44, *p* = 0.075).

## 4. Discussion

In this study, unsupervised profiling of circulating T-cell subpopulations identified three distinct post-transplant recipient clusters characterized by differential representation of CD4^+^, CD8^+^, and innate-like cytotoxic T-cell compartments. These cluster-defined profiles were associated with differences in longitudinal graft function trajectories during the first post-transplant year. Because the clusters were derived from immunophenotyping performed at 12 months after transplantation, they are best interpreted as reflecting stabilized patterns of post-transplant immune phenotypic patterns rather than predictors of subsequent outcomes. Accordingly, the observed associations should be interpreted as retrospective and hypothesis generating rather than causal or predictive. Importantly, this study applies a data-driven immune stratification approach to characterize multidimensional T-cell configurations in kidney transplant recipients and relate them to graft functional trajectories.

The CD8-skewed cluster was characterized by enrichment of differentiated CD8^+^CD28^−^ T cells together with relative reduction of CD4^+^ compartments. Loss of CD28 expression is a hallmark of advanced T-cell differentiation and replicative senescence resulting from repeated antigenic stimulation. These CD8^+^CD28^−^ T cells typically display reduced proliferative capacity but enhanced cytotoxic activity and pro-inflammatory cytokine production. Expansion of such populations has been described in aging individuals and transplant recipients exposed to persistent antigenic stimulation, including chronic viral infections. In kidney transplantation, accumulation of differentiated CD28^−^ T cells has been associated with altered immune regulation and graft outcomes, suggesting that these cells reflect long-term immune phenotypic patterns rather than acute alloimmune activation [12,25,26,27,28].

The association between this cluster, recipient age, and CMV seropositivity further supports the concept that post-transplant immune configurations reflect cumulative immunological history. CMV infection is a major driver of long-term phenotypic patterns of the T-cell compartment. Chronic or latent CMV exposure promotes sustained antigenic stimulation and expansion of highly differentiated CD8^+^ T cells, many of which lose CD28 expression and acquire terminal effector phenotypes, a process often referred to as memory inflation. This mechanism contributes to the accumulation of cytotoxic CD28-negative T cells in aging individuals and transplant recipients and has been implicated in shaping long-term immune adaptation after transplantation [12,25,26,27,28,29]. In parallel, age-associated immune phenotypic patterns (immunosenescence) are characterized by contraction of naïve T-cell pools and expansion of highly differentiated CD8^+^ effector populations [29]. The association between recipient age and the CD8-skewed cluster in our cohort, therefore, aligns with previous observations linking aging to expansion of cytotoxic and senescent T-cell phenotypes in transplant populations [25,26,27,28,29].

While the CD8-skewed cluster reflects differentiation of the adaptive cytotoxic T-cell compartment, a second cluster in our cohort highlighted the potential contribution of cytotoxic populations bridging innate and adaptive immunity.

The innate-like cytotoxic cluster identified in our analysis was enriched in CD3^+^CD16^+^/56^+^ T cells, a population often described as NK-like or NKT-like T cells. These cells express conventional T-cell receptors while also displaying NK-associated receptors such as CD16 and CD56, thereby bridging innate and adaptive immunity. Functionally, they can mediate rapid cytotoxic responses, antibody-dependent cellular cytotoxicity, and cytokine production independent of classical antigen presentation Increasing evidence suggests that NK-related immune mechanisms contribute to kidney allograft injury, including antibody-independent pathways of chronic graft dysfunction. High-dimensional immune profiling studies have linked NK-related immune signatures with graft dysfunction even in the absence of overt rejection [14,15,30,31,32]. Although this cluster was not associated with higher rejection frequency in the present cohort, enrichment of CD16^+^/56^+^ cytotoxic populations may reflect broader immune phenotypic patterns associating with differing graft functional trajectories.

From a clinical perspective, the identified clusters in this study were associated with differences in graft function trajectories during the first post-transplant year. Although cross-sectional graft function differences at 12 months were not statistically significant, longitudinal modeling revealed distinct functional patterns across clusters. These findings suggest that circulating immune configurations may capture aspects of post-transplant immune adaptation not fully reflected by conventional clinical parameters. However, these patterns should not be interpreted as discrete pathogenic mechanisms or validated clinical phenotypes. Rather, they likely represent cohort-specific summaries of integrated immune environments shaped by host characteristics, viral exposure, and T-cell differentiation processes.

The principal contribution of the present study is therefore not the identification of new immune entities but the integrative characterization of multidimensional T-cell composition patterns and their clinical correlates in a transplant cohort. Such clustering approaches may help generate testable hypotheses for future mechanistic and biomarker studies.

Several limitations should be acknowledged. Immune profiling was performed at a single post-transplant time point in a single-center cohort, precluding causal inference and limiting generalizability. In addition, the identified clusters were derived from an unsupervised single-cohort analysis and should therefore be considered exploratory patterns requiring external validation.

Cluster robustness was supported by concordance between Ward hierarchical clustering and k-means clustering; however, formal bootstrap resampling and leave-one-feature-out sensitivity analyses were not performed. These approaches should be considered in future validation studies.

Detailed data on CMV reactivation or viral load during follow-up were not available, and the observed association with CMV serostatus should therefore be interpreted as reflecting prior viral exposure rather than active infection. The immunophenotyping panel focused primarily on differentiation markers and did not include activation or exhaustion markers such as HLA-DR, PD-1, or CCR7/CD45RA, which could further characterize functional T-cell states. Future studies integrating broader immune profiling, functional assays, and tissue-level analyses may provide deeper insight into post-transplant immune remodeling.

In conclusion, this exploratory analysis identified distinct cluster-defined post-transplant T-cell composition patterns associated with differences in graft function trajectories during the first year after kidney transplantation. These findings support the potential value of multidimensional immune profiling for characterizing post-transplant immune heterogeneity. Longitudinal and external validation studies are required to determine clinical relevance.

## Figures and Tables

**Table 1:** Baseline clinical and transplant-related characteristics of the study population (n = 112). Values are presented as median (interquartile range) for continuous variables and number (percentage) for categorical variables.

Recipient’s Characteristics	Overall Cohort (n = 112)
Age, years	48.5 (39–57)
Male sex, n (%)	77 (68.7)
Female sex, n (%)	35 (31.3)
Primary kidney disease	
— Nephrosclerosis/hypertension	6 (5.3)
— Primary glomerulopathies	26 (23.2)
─ Diabetes mellitus	6 (5.3)
— Urinary tract disease/stones	6 (5.3)
─ Reflux nephropathy	13 (11.6)
— Polycystic kidney disease	24 (21.4)
— Other	16 (14.2)
— Unknown	15 (13.3)
Dialysis vintage, months	87 (34–127)
Pre-emptive transplantation, n (%)	12 (10.7)
Cold ischemia time, h	17 (0–19)
Delayed graft function, n (%)	20 (17.9)
Rejection episode, n (%)	10 (8.9)
Donor type	
— Deceased donor	83 (74.1)
— Living donor	29 (25.9)
Induction therapy	
— Basiliximab	95 (84.8)
— ATG	17 (15.2)

**Table 2:** Quantitative differences in circulating T-cell subpopulations across T12 post-transplant T-cell clusters. Frequencies of circulating T-cell subpopulations at 12 months post-transplantation are presented as median (interquartile range). Comparisons across clusters were performed using the Kruskal–Wallis test. Values are presented as medians (interquartile range).

T-Cell Subpopulation (% Parent Population)	CD8-Skewed Cluster (n = 30)	Innate-like Cluster (n = 18)	CD4-Dominant Cluster (n = 64)	*p*-Value
CD3^+^	80.0 (74.9–85.9)	60.5 (48.2–71.2)	81.2 (74.8–86.0)	0.00023
CD3^+^CD4^+^	36.9 (29.6–45.4)	41.2 (34.2–54.4)	50.6 (44.9–55.0)	<0.001
CD3^+^CD8^+^	43.5 (37.1–51.1)	13.7 (0.0–22.5)	27.3 (23.3–30.4)	<0.001
CD3^+^CD4^+^CD28^+^	30.3 (23.1–39.3)	30.7 (6.7–39.0)	47.3 (43.2–52.9)	<0.001
CD3^+^CD4^+^CD28^−^	3.8 (1.7–9.1)	2.1 (0.0–5.2)	1.4 (0.6–3.1)	0.009
CD3^+^CD8^+^CD28^+^	14.7 (10.6–18.5)	7.1 (0.0–11.1)	18.5 (15.2–21.5)	<0.001
CD3^+^CD8^+^CD28^−^	28.5 (21.9–36.0)	3.8 (0.0–9.3)	7.8 (4.5–11.4)	<0.001
CD3^+^CD16^+^/56^+^	9.5 (6.3–25.4)	27.4 (6.4–39.2)	8.3 (5.6–14.1)	0.048
CD3^+^CD4^+^CD25^+^FoxP3	3.9 (3.4–4.8)	4.5 (2.1–5.8)	4.6 (3.1–5.7)	0.352

**Table 3:** Clinical and transplant-related characteristics across post-transplant T-cell clusters at 12 months. Values are presented as median (interquartile range) or number (percentage). Comparisons across clusters were performed using Kruskal–Wallis, chi-square tests, or Fisher’s exact test as appropriate.

CD4-Dominant Cluster (n = 64)	*p*-Value
45.0 (36.5–55.0)	0.039
84.0 (18.3–124.5)	0.338
16.0 (0.0–19.0)	0.645
1.19 (1.00–1.34)	0.185
63.7 (54.3–77.8)	0.192
44 (68.8)	0.324
47 (73.4)	0.927
17 (26.6)	
9 (14.1)	0.327
6 (9.4)	0.287

**Table 4:** Exploratory multinomial logistic regression analysis of clinical variables associated with post-transplant T-cell cluster assignment. Multinomial logistic regression analysis evaluating clinical variables associated with cluster assignment among 112 kidney transplant recipients. Cluster assignment was the dependent variable, with the CD4-dominant cluster as the reference category. Results are presented as odds ratios (ORs) with 95% confidence intervals (CIs). Covariates were prespecified based on clinical relevance and biological plausibility, including recipient age, sex, donor type, dialysis vintage, ATG induction therapy, and baseline CMV IgG serostatus. Statistical significance was defined as a two-sided *p*-value < 0.05. Abbreviations: OR, odds ratio; CI, confidence interval; ATG, anti-thymocyte globulin; CMV, cytomegalovirus.

Variable	CD8-Skewed vs. CD4-Dominant OR (95% CI)	*p*-Value	Innate-like vs. CD4-Dominant OR (95% CI)	*p*-Value
Recipient age (per year)	1.05 (1.01–1.10)	0.018	1.03 (0.99–1.08)	0.161
Male sex	1.37 (0.47–3.98)	0.565	0.67 (0.21–2.07)	0.483
Deceased donor	0.28 (0.05–1.46)	0.131	0.74 (0.12–4.50)	0.748
Dialysis vintage (per month)	1.01 (1.00–1.02)	0.195	1.00 (0.99–1.02)	0.834
ATG induction	0.86 (0.32–2.27)	0.756	2.91 (0.90–9.44)	0.075
CMV IgG positive at baseline	7.60 (1.67–34.60)	0.009	0.78 (0.08–8.09)	0.836

## Data Availability

Data are contained within the article and/or the Supplementary Materials.

## Supplementary Materials

The following supporting information can be downloaded at: https://www.mdpi.com/article/10.3390/medsci14020238/s1. Table S1: Silhouette coefficient for k = 2–5 cluster solutions using Ward hierarchical clustering; Table S2: Concordance between Ward hierarchical clustering and k-means clustering (k = 3); Figure S1: Silhouette coefficients for k = 2–5 cluster solutions using Ward clustering; Figure S2: Hierarchical clustering dendrogram of standardized T-cell subset frequencies using Ward linkage.
