# Supplementary material for: Unsupervised Immune Profiling Identifies Distinct Post-Transplant T-Cell Clusters Associated with Kidney Allograft Function
Source: Med Sci (Basel). 2026 May 4;14(2):238. doi: 10.3390/medsci14020238 (PMC13214605; doi:10.3390/medsci14020238)
Supplement: Supplementary file 1 [file medsci-14-00238-s001.zip › medsci-4264239-supplementary.pdf]

Supplementary Methods – Robustness analysis using k-means clustering

As a robustness analysis, k-means clustering was applied to the same standardized (z-score transformed) T-cell subset frequencies used for the primary Ward hierarchical clustering. Standardization was performed to minimize the influence of differences in variable scale and variance across immunophenotypic features. To ensure direct comparability with the primary analysis, the number of clusters was fixed at **k = 3**, corresponding to the optimal solution identified by silhouette analysis in the primary Ward approach. Silhouette coefficients were evaluated for k = 2–5 cluster solutions, with the highest value observed for k = 3. K means clustering was implemented using Euclidean distance with random initialization and multiple starting configurations to ensure solution stability. Concordance between Ward and k-means cluster assignments was evaluated descriptively using a cross-tabulation of cluster membership. Key downstream findings (PCA visualisation and longitudinal graft function models) were re-evaluated using k-means derived immune states to confirm consistency of the primary findings. Formal bootstrap resampling or feature-selection sensitivity analyses were not performed and should be considered future validation steps. Given the exploratory design and modest cohort size, concordance across complementary clustering approaches was used as a pragmatic internal robustness assessment.

Table S1: Silhouette coefficient for k=2-5 cluster solutions using Ward hierarchical clustering

k	Silhouette	Cluster sizes
**2**	0.206	82 / 30
**3**	**0.225 (highest)**	**30 / 18 / 64**
4	0.207	18 / 27 / 64 / 3
5	0.224	64 / 27 / 7 / 3 / 11

The highest silhouette coefficient supported the three-cluster solution used in the primary analysis.

Table S2: Concordance between Ward hierarchical clustering and k-means clustering (k = 3).
Cross-tabulation of cluster membership between the primary Ward hierarchical clustering and the k-means robustness analysis performed using the same standardized T-cell subset frequencies. Agreement between clustering approaches supports the stability of the identified immune states.

Ward \ k-means	Cluster 0	Cluster 1	Cluster 2
Cluster 0	18	9	3
Cluster 1	5	12	1
Cluster 2	1	63	0

Overall agreement (row-wise maximum matching): 83.04%

This concordance indicates that the principal cluster structure was reproducible across two different unsupervised methods.

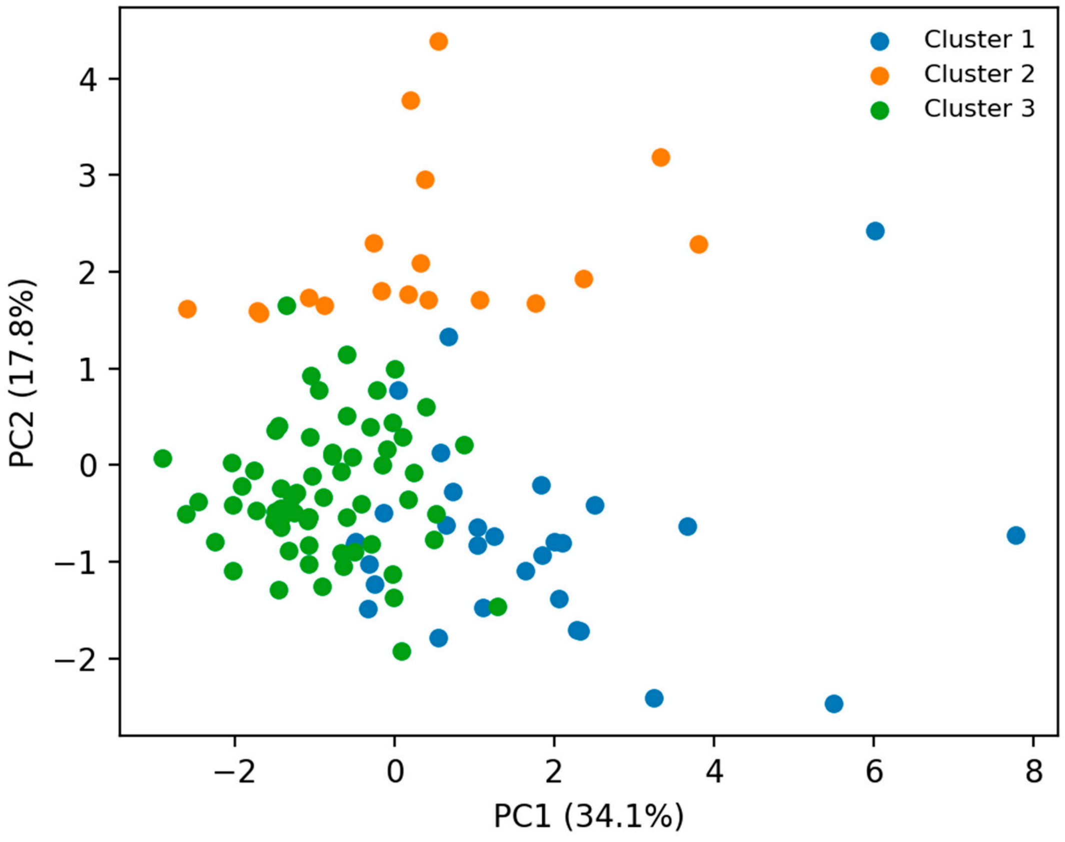

Figure S1: Silhouette coefficients for k = 2–5 cluster solutions using Ward clustering. The highest silhouette value was observed for k = 3, supporting the selected primary solution.

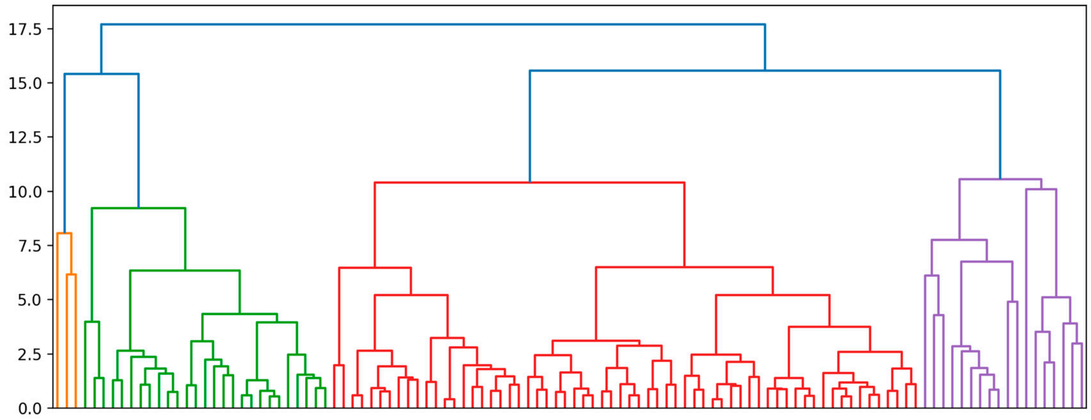

Figure S2: Hierarchical clustering dendrogram of standardized T-cell subset frequencies using Ward linkage.
